# Artificial Intelligence Based Methods for Asphaltenes Adsorption by Nanocomposites: Application of Group Method of Data Handling, Least Squares Support Vector Machine, and Artificial Neural Networks

**DOI:** 10.3390/nano10050890

**Published:** 2020-05-06

**Authors:** Mohammad Sadegh Mazloom, Farzaneh Rezaei, Abdolhossein Hemmati-Sarapardeh, Maen M. Husein, Sohrab Zendehboudi, Amin Bemani

**Affiliations:** 1 Department of Petroleum Engineering, Petroleum University of Technology, Ahwaz 61991-71183, Iran; sadeghmazloom1993@gmail.com (M.S.M.); aminbemani90@yahoo.com (A.B.); 2 Department of Petroleum Engineering, Shahid Bahonar University of Kerman, Kerman 7616913439, Iran; rezaei.f1373@gmail.com; 3 College of Construction Engineering, Jilin University, Changchun 130600, China; 4 Department of Chemical & Petroleum Engineering, University of Calgary, Calgary, AB T2N 1N4, Canada; 5 Department of Process Engineering, Memorial University, St. John’s, NL A1C 5S7, Canada; szendehboudi@mun.ca

**Keywords:** asphaltene, nanocomposite, artificial intelligence, adsorption, statistical analysis, deposition

## Abstract

Asphaltenes deposition is considered a serious production problem. The literature does not include enough comprehensive studies on adsorption phenomenon involved in asphaltenes deposition utilizing inhibitors. In addition, effective protocols on handling asphaltenes deposition are still lacking. In this study, three efficient artificial intelligent models including group method of data handling (GMDH), least squares support vector machine (LSSVM), and artificial neural network (ANN) are proposed for estimating asphaltenes adsorption onto NiO/SAPO-5, NiO/ZSM-5, and NiO/AlPO-5 nanocomposites based on a databank of 252 points. Variables influencing asphaltenes adsorption include pH, temperature, amount of nanocomposites over asphaltenes initial concentration (D/C_0_), and nanocomposites characteristics such as BET surface area and volume of micropores. The models are also optimized using nine optimization techniques, namely coupled simulated annealing (CSA), genetic algorithm (GA), Bayesian regularization (BR), scaled conjugate gradient (SCG), ant colony optimization (ACO), Levenberg–Marquardt (LM), imperialistic competitive algorithm (ICA), conjugate gradient with Fletcher-Reeves updates (CGF), and particle swarm optimization (PSO). According to the statistical analysis, the proposed RBF-ACO and LSSVM-CSA are the most accurate approaches that can predict asphaltenes adsorption with average absolute percent relative errors of 0.892% and 0.94%, respectively. The sensitivity analysis shows that temperature has the most impact on asphaltenes adsorption from model oil solutions.

## 1. Introduction

Depending on oil composition and process conditions, asphaltenes deposition may pose a serious concern during the production of light and heavy oils [1]. Based on solubility criteria, asphaltenes are fractions of the crude oil that are insoluble in low molecular weight (MW) paraffins, whilst soluble in light aromatics such as pyridine, benzene, and toluene [2]. Asphaltenes are the heaviest and most polar components of crude oil [3,4]. Using laser-induced breakdown spectroscopy (LIBS), three main metals were found; namely V, Ni, and Fe, and traces of ten other metals such as Mo, Cu, P, Mn, Cd, Si, Co, Ti, Pb, and Ca were detected in asphaltenes [5]. The physical and chemical nature, and amount of asphaltenes extracted from a crude oil depend on many factors (e.g., solvent, contact time, dilution proportion, extraction procedure, and temperature) [6,7,8]. Such a disparity among the characteristics of asphaltenes molecules causes a challenge when dealing with these complicated molecules. Furthermore, asphaltenes molecules tend to aggregate, leading to significant speculation on their MW and size [9]. Several researchers proposed an average MW of monomer asphaltenes of 1000 g/mol, using various techniques [10,11,12,13,14]. H-bonding as well as π−π stacking are important intramolecular interactions for asphaltenes aggregation [15,16]. Aggregation, in turn, induces asphaltenes deposition [17]. In addition, heteroatoms, particularly those containing polar moieties (e.g., pyridinic, phenolic, and carboxylic), are vital for asphaltenes adsorption onto surfaces [18,19]. Functional groups including N and O heteroatoms, and to a minor extent S, furnish asphaltenes with active surface properties instigating asphaltenes surface adsorption [20]. Polar interactions are the major contributors to asphaltenes adsorption [21]. Asphaltenes self-association and adsorption result in a number of problems during crude oil production and upgrading; including pipeline plugging, wettability alteration, pore blockage, and catalyst coking [22,23,24,25,26].

The literature studies suggest different approaches to overcome issues induced by asphaltenes self-association. For instance, asphaltene inhibitors (e.g., nonionic surfactants and plant liquid elicited from cashew-nut shells) [27,28,29] and dispersants such as nonpolymeric surfactants offer effective solutions to deferred production [30,31]. Other techniques such as mechanical, chemical, bacterial, thermal, and ultrasonic treatments have been applied to clean deposited asphaltenes [32,33,34,35,36,37,38,39]. It should be noted that asphaltenes can reprecipitate due to changes in thermodynamic conditions such as pressure and temperature during production, which may render production uneconomical [40]. 

Nanoparticles (NPs) have shown potential application over a wide range of facilities, from oil production to surface and refining facilities, due to their small size, proper mobility within porous media, surface area to volume ratio, and catalytic activities [41,42]. For instance, NPs can be applied to promote asphaltenes adsorption, oxidation, gasification, and in situ combustion (ISC) processes [43,44,45,46,47,48]. Moreover, NP coatings have been employed to prevent pipeline scaling and corrosion issues [49,50]. A field test in Colombia exhibited successful use of NPs to increase production [51]. However, an adequate number of pilot projects should be conducted before drawing reliable conclusions on the efficiency of NPs and nanocomposites deployment, especially under the harsh reservoir conditions. Prior to NP use in oil fields, numerous experimental investigations have resulted in interesting findings while employing NPs to solve asphaltene-related problems. For example, Madhi et al. used SiO_2_, Al_2_O_3_, and MgO NPs to adsorb asphaltenes from toluene model oils [52]; it was concluded that SiO_2_ particles are more effective. Different adsorption models; including Dubinin–Radushkevich, Langmuir, Temkin, and Freundlich, were used to investigate the mechanism of asphaltenes adsorption [52]. Based on asphaltenes adsorption from toluene model solutions, NPs of TiO_2_, MgO, CaO, Fe_3_O_4_, Co_3_O_4_, NiO as well as different sizes of nickel were deemed applicable to asphaltenes adsorption from oil [53,54,55]. The effects of initial asphaltene concentration, contact time, and mass and size of NPs on asphaltene adsorption were studied [53,54,55]. In situ prepared NPs within crude oil displayed much higher adsorption capacity than commercial NPs [56,57]. Other studies, using toluene model solutions, demonstrated that NPs with acidic surface properties adsorb higher amounts of asphaltenes, compared to neutral and basic NPs [58,59]. Moreover, NPs displayed more affinity toward asphaltenes adsorption from model toluene solutions in the presence of resins [60]. The use of NiO/SAPO-5 for asphaltenes adsorption from toluene model oils has been investigated with detailed data and information using Brunauer–Emmett–Teller (BET), X-ray diffraction (XRD), transmission electron microscopy (TEM), and Fourier-transform infrared spectroscopy (FTIR) [61]. The response surface method was implemented to maximize asphaltenes adsorption [61]. The resultant model was used to determine the performance of NiO/ZSM-5 nanocomposites and study the economic feasibility of asphaltenes adsorption onto nanocomposites [62]. Mohammadi et al. synthesized NiO/AlPO-5 and NiO/ZSM-5 to investigate asphaltenes adsorption under various conditions; including pH, amount of NPs over asphaltenes initial concentration (D/C_0_), and temperature [63]. Asphaltenes adsorption onto these particles was modeled using adaptive neuro-fuzzy interference system called ANFIS [63].

In this work, asphaltenes adsorption from model solutions using different nanocomposites is modeled at various temperatures, pH, and with different amounts of nanocomposites with varying physicochemical properties; including total surface area and pore volumes. It should be noted that pH value is attributed to the solution of nanocomposites (adsorbent), base, and acid before adding the model oil (asphaltene + toluene). pH is adjusted by adding acids (e.g., citric acid) and base (e.g., ethylenediamine) [61]. Different machine learning protocols; including least squares support vector machine (LSSVM), artificial neural network (ANN), and group method of data handling (GMDH) are utilized to predict the adsorption at various process and thermodynamic conditions. Furthermore, various optimization methods; namely Levenberg–Marquardt (LM), Bayesian regularization (BR) algorithm, conjugate gradient with Fletcher-Reeves updates (CGF), scaled conjugate gradient (SCG) approach, genetic algorithm (GA), particle swarm optimization (PSO), coupled simulated annealing (CSA), imperialistic competitive algorithm (ICA), and ant colony optimization (ACO) are implemented to obtain the optimal values of the model parameters. The precision and reliability of the collected data are assessed as a first step toward developing proper models. To the best of our knowledge, this is the first time that asphaltenes adsorption by three nanocomposites; namely NiO/ZSM-5, NiO/SAPO-5 and NiO/AlPO-5, are modeled using three connectionist modeling protocols and optimized by nine procedures. Furthermore, the quality of the experimental data is evaluated on the basis of common statistical parameters. The findings of this study can help to better understand the effective parameters impacting asphaltenes adsorption and to design and operate effective asphaltene removal techniques.

## 2. Theory and Methods 

### 2.1. Experimental Dataset

In order to develop reliable models based on LSSVM, ANN, and GMDH for predicting asphaltenes adsorption from oils using nanocomposites, a comprehensive experimental adsorption data pertaining to NiO/ZSM-5, NiO/SAPO-5 and NiO/AlPO-5 at various temperatures, pH values, and amounts of nanocomposites were collected from the literature. This set of experimental data consists of 252 points under different operational conditions, as detailed in the literature [61,62,64]. Nanocomposites properties and the experimental conditions are listed in Table 1 and Table 2, respectively.

### 2.2. Models and Procedures

#### 2.2.1. Least Squares Support Vector Machine (LSSVM)

Support vector machine (SVM) is a conventional machine learning approach. Generally, machine learning performs data classification and minimizes structural risk by simplification of high dimensional space and implementing kernel function, as shown in Equation (1). The modified version of SVM approach; namely least squares support vector machine (LSSVM), uses least-squares results in the form of a principle to obtain the minimum structural risk [65,66,67]. Thus, the fundamental equation of LSSVM can be expressed as follows:

(1) $$min_{\omega ,b,e}J\left(\omega ,e\right)=\frac{1}{2}\omega^{2}+\gamma \overset{m}{\underset{i=1}{\sum }}X_{i}$$

such that $Y_{i}\left(\omega^{\prime }X_{i}+b\right)+\varepsilon_{i}\geq 1$; $\varepsilon_{i}\geq 0$; *i* = 1, 2, …, m

(2) $$min_{\omega ,b,e}J\left(\omega ,e\right)=\frac{1}{2}||\omega^{2}||+\frac{1}{2}\gamma \overset{N}{\underset{k=1}{\sum }}e_{k}^{2}$$

such that $Y_{i}=\omega^{T}\varphi \left(X_{i}\right)+b+e_{i}$; *i* = 1, 2, …, m

where *J*, *X_i_,* and *Y_i_* resemble the risk bound, slack variable, and binary target, respectively. γ, ω, *b*, $\varepsilon_{i},\;\varphi \left(X_{i}\right),$ and $e_{i}$ stand for the regularization parameter, weight matrix, bias, slack variable, kernel function, and error, respectively. To solve this problem, the Lagrangian function is determined as follows:

(3) $$L_{LSSVM}=\frac{1}{2}||w^{2}||+\frac{1}{2}\gamma \overset{N}{\underset{i=1}{\sum }}e_{k}^{2}-\overset{N}{\underset{k=1}{\sum }}\alpha_{k}\left\{\left(\omega .\emptyset \left(x_{k}\right)\right)+b+e_{k}-y_{k}\right\}$$

In Equation (3), $\alpha_{k}$ represents the Lagrangian multipliers. The derivatives of Equation (3) in terms of *ω*, *b*, *e,* and $\alpha_{k}$ are obtained by Equation (4), which is used to determine the parameters:

(4) $$\frac{\partial L_{LSSVM}}{\partial w}=\frac{\partial L_{LSSVM}}{\partial b}=\frac{\partial L_{LSSVM}}{\partial e_{k}}=\frac{\partial L_{LSSVM}}{\partial \alpha_{k}}=0$$

(5) $$w=\overset{N}{\underset{k=1}{\sum }}\alpha_{k}\emptyset \left(x_{k}\right)$$

(6) $$\overset{N}{\underset{k=1}{\sum }}\alpha_{k}=0$$

(7) $$\alpha_{k}=\gamma e_{k}\;k=1,\ldots ,N$$

(8) $$\left(w.\emptyset \left(x_{k}\right)\right)+b+e_{k}-y_{k}=0\;k=1,\ldots N$$

Following the above equations, a linear function system is defined as given below:

(9) $$\left[\begin{array}{cc} 0 & I_{N}^{T}\\ I_{N} & \Omega +\gamma^{-1}l_{N} \end{array}\right]\left[\begin{array}{c} b\\ \alpha \end{array}\right]=\left[\begin{array}{c} 0\\ Y \end{array}\right]$$

where *Y = [y*_1_*,…y_N_]*, *l_v_ = [1,…l]* and *α=[α*_1_*, …, α_N_]*. $\Omega_{ij}$, kernel function, can be formulated by the following equation [68]:

(10) $$\Omega_{ij}=\emptyset \left(x_{i}\right)\emptyset \left(x_{j}\right)=K\left(x_{i},\;x_{j}\right)$$

In the current study, the radial basis function is selected as a kernel function for the LSSVM algorithm. 

#### 2.2.2. Artificial Neural Network (ANN)

One of the popular branches of computational-based modeling is the artificial neural network (ANN), which is constructed on the basis of biological nervous systems. ANN effectively explores patterns within the data and creates new relationships between the target value and the important variables in the system. ANNs consist of a huge number of interconnected elements known as neurons [69,70]. Neurons act as processing units and are organized in various layers. Neurons are used for pattern recognition, clustering, function approximation, and classification. The radial basis function (RBF) and multilayer perceptron (MLP) neural networks are prominent forms of ANNs. It is worth noting that the main difference among these networks is the procedure neurons perform. RBF-ANN is constructed based on an output layer, a hidden layer, and an input layer. The hidden layer has neurons, which contain a radial basis function for their activation functions. Implementing linear optimization approach, this algorithm can find the best solution by adjusting weights during mean square error minimization. The output for the input pattern of “*x*” can be obtained using the following relationship [71]:

(11) $$y_{i}\left(x\right)=\overset{m}{\underset{i=1}{\sum }}w_{i}\emptyset_{i}\left(\left|\left|x-x_{i}\right|\right|\right)$$

where *w_i_* and $\emptyset_{i}$ denote the connection weight and radial basis function, respectively. There are different types of radial basis functions (e.g., Gaussian function), given below:

(12) $$\emptyset \left(\left|\left|x_{i}-x_{j}\right|\right|\right)=\exp\left(-\frac{{\left|\left|x-x_{i}\right|\right|}^{2}}{2\sigma^{2}}\right)$$

In Equation (12), $x_{j}$ and *σ* refer to the center of function and the Gaussian spread, respectively. 

As stated previously, MLP is known as the other form of ANN. This algorithm has several layers with the first one being the input layer and the last one being the output layer. The input and output layers are connected by intermediate and hidden layers. In the hidden and output layers, different forms of activation functions can be applied; including:

(13) $$Sigmoid=\frac{1}{1+e^{-x}}$$

(14) Linear=Purelin=x

(15) $$ArcTan=tan^{-1}\left(x\right)$$

(16) $$Sinusid=\sin\left(x\right)$$

(17) $$Tansig=Tanh=\frac{2}{1+e^{-2x}}-1$$

(18) Binary Step : x for x<0 and−x for x≥0

**Figure 1:**
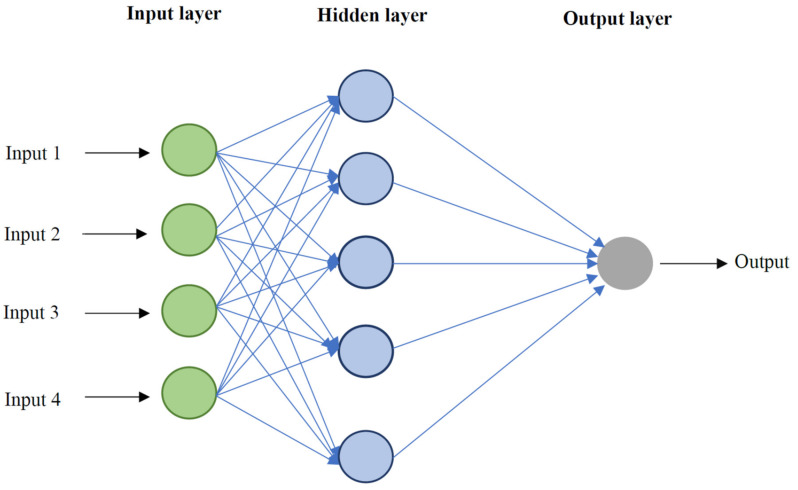
Simple schematic diagram of multilayer perceptron—artificial neural network (MLP-ANN) algorithm used in this study.

By considering an MLP model with two hidden layers, tansig and logsig activation functions for the hidden layers, respectively, and purlin for the output layer, the output can be calculated as follows:

(19) $$Output=purelin\left(w_{3}\times \left(\;Sigmoid\left(w_{2}\times \left(Tansig\left(x\right)+b_{1}\right)\right)+b_{2}\right)+b_{3}\right)$$

where *b*_1_ and *b*_2_ introduce the first and second hidden layer bias vectors and b_3_ resembles the output layer bias vector, accordingly. In addition, *w*_1_ and *w*_2_ represent the first and second hidden layers’ weight matrixes, respectively, and *w*_3_ is the output layer weight matrix. In this study, optimization algorithms, namely CGF, SCG, BR, and LM are employed to enhance the performance of MLP model. A schematic of the MLP-ANN algorithm is depicted in Figure 1.

#### 2.2.3. Group Method of Data Handling (GMDH)

This method was proposed by Shankar as a self-organizing system [72]. GMDH was later used for pattern recognition, artificial intelligence, regression analysis [73,74], acoustic and seismic analysis, microprocessor-based hardware, multisensor signal processing, weather modeling, medical diagnostics, and prediction and classification in various engineering and science disciplines such as chemical engineering, petroleum engineering, mechanical engineering, and environmental engineering [74,75,76,77]. GMDH, also called polynomial neural network (PNN), is constructed based on layered structure. This structure has independent neurons, which are coupled by means of quadratic polynomials. Initially, Ivankhnenko proposed GMDH based on the optimum selection of quadratic polynomial formulations [78]. To predict the relationship between outputs and inputs, Volterra-Kolmogorov-Gabor series is utilized as shown below:

(20) $$Y_{i}=a+\overset{M}{\underset{i=1}{\sum }}b_{i}x_{i}+\overset{M}{\underset{i=1}{\sum }}\overset{M}{\underset{j=1}{\sum }}C_{ij}x_{i}x_{j}+\ldots \overset{M}{\underset{i=1}{\sum }}\overset{M}{\underset{j=1}{\sum }}\ldots \overset{M}{\underset{k=1}{\sum }}d_{ij\ldots k}x_{i}x_{j}\ldots x_{k}$$

where *x_i_* and *Y_i_* introduce the inputs and outputs; *M* refers to the number of independent parameters; and *a*, *b_i_*, *c_ij_*, and *d_ij_*_…*k*_ denote the polynomial coefficients. Two independent parameters are then coupled together by a quadratic polynomial formulation and new parameters, *Z*_1_,..*Z_n_*, to replace the former values. The quadratic polynomials can be written as follows:

(21) $$Z_{i}^{GMDH}=ax_{i}+bx_{j}+Cx_{i}x_{j}+dx_{i}^{2}+ex_{j}^{2}+f$$

The new matrix is expressed by $v_{z}=\left(z_{1},\ldots ,z_{n}\right)$. To determine the coefficients of Equation (21), the least square method is used. This method minimizes the sum of the squared deviations between real and predicted values as follows:

(22) $$\delta_{j}^{2}=\sum_{i=1}^{N_{t}}{\left(y_{i}-z_{i}^{GMDH}\right)}^{2}\;\;\mathrm{where}\;j=1,2,\ldots ,\left(\begin{array}{c} M\\ 2 \end{array}\right)$$

(23) $$Y=A^{T}X$$

in which, *A = {a,b,c,d,e,f}* denotes the quadratic polynomial coefficient vector and *T* stands for the transposed matrix. Finally, the least square method leads to the following solution:

(24) $$A^{T}=YX^{T}{\left(XX^{T}\right)}^{-1}$$

**Figure 2:**
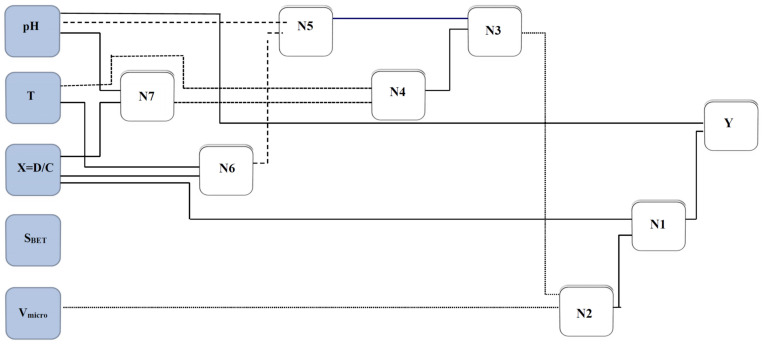
A simplified structure of the presented group method of data handling (GMDH) for estimating asphaltenes adsorption.

A schematic of the GMDH model proposed in this study is illustrated in Figure 2. As it is clear from Figure 2, the designed network has an input layer, seven middle layers, and an output layer. The genome and nodal formulation of this network can be determined by the expressions given in Table 3.

### 2.3. Optimization Approaches

In order to optimize the models applied in this study, nine optimization procedures are used. The employed optimization techniques include particle swarm optimization (PSO), imperialistic competitive algorithm (ICA), ant colony optimization (ACO), conjugate gradient with Fletcher-Reeves updates (CGF), Levenberg–Marquardt (LM), coupled simulated annealing (CSA), Bayesian regularization (BR) algorithm, genetic algorithm (GA), and scaled conjugate gradient (SCG). For MLP optimization, CGF, SCG, BR, and LM are used. For more details about optimization methods of MLP models, readers can visit the literature [79,80,81,82,83,84,85,86].

#### 2.3.1. Genetic Algorithm

**Figure 3:**
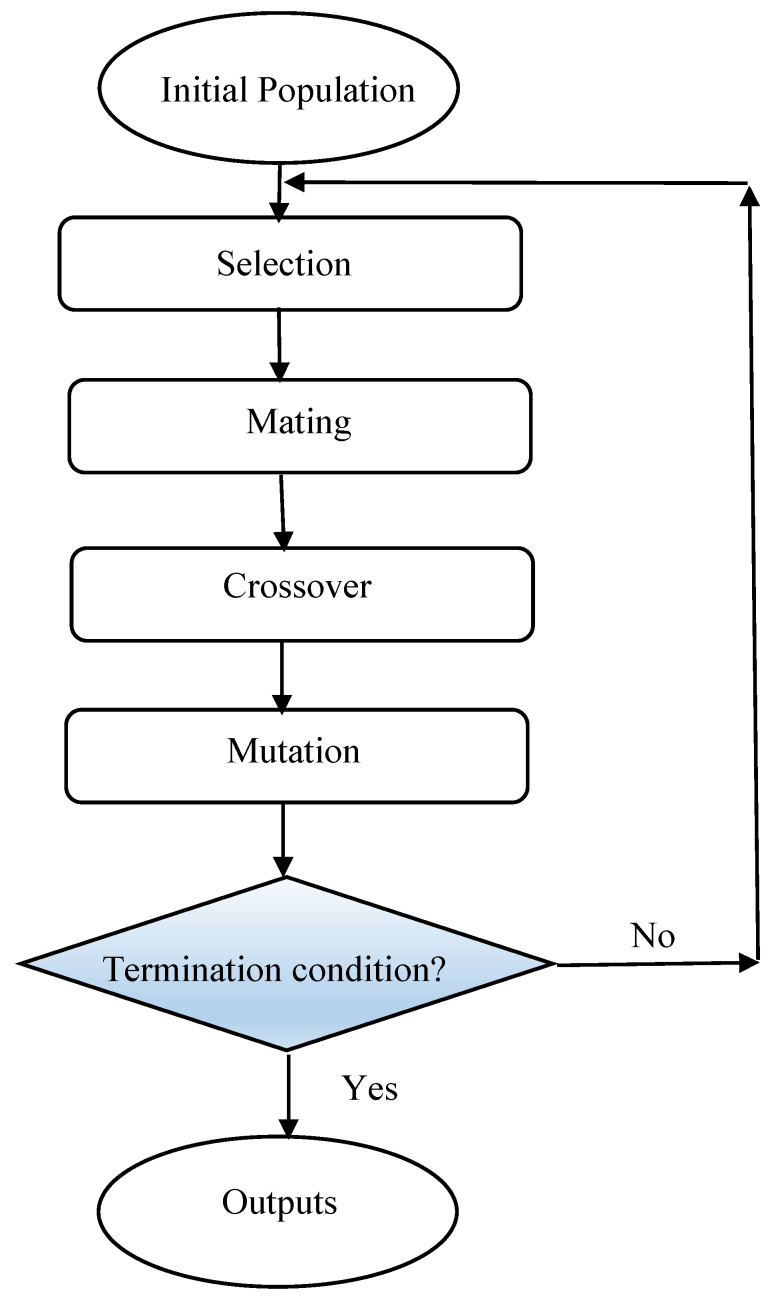
Flowchart of genetic algorithm (GA) optimization method.

A metaheuristic algorithm named genetic algorithm (GA) was inspired based on the natural selection process. GAs use operators; including selection, crossover, and mutation, in their search and optimization problem. In this algorithm, probable solutions called population which contains individuals or creatures, move toward the optimum solutions. First, the population is produced randomly; then according to the obtained fitness values for each member of a population, the best individuals are selected to make the future population by considering crossover and mutation effects on them. The previous population is replaced by a new population and the process continues until the algorithm reaches satisfactory accuracy or maximum number of iterations [87,88]. A brief procedure of GA optimization is depicted in Figure 3. 

#### 2.3.2. Particle Swarm Optimization

**Figure 4:**
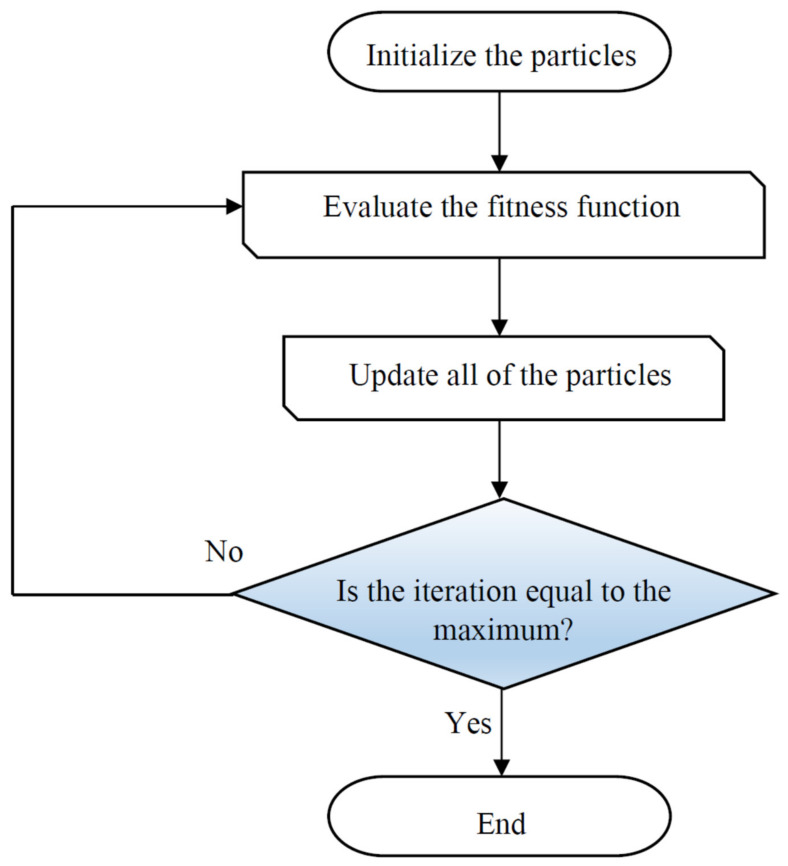
Schematic of the main steps used in the particle swarm optimization (PSO) algorithm.

Particle swarm optimization (PSO) [89] was constructed by Kennedy based on natural flocking and swarming of birds and insects. The first step of PSO is generation of a population of random solutions, known as particles. This population moves through the problem space based on the present best particles. The main characteristics of a particle are the position and velocity, which are used for searching through space with an appropriate value of fitness. It is worth noting that each particle saves two dominant pieces of information including the best global position (*g_best_*) and the best visited position (*p_best_*) [90]. The algorithm has an iterative performance so that the obtained solution for each iteration is compared with the global best and self-local best particle. The next position of a particle can be determined as follows:

(25) $$v_{i}\left(t+1\right)=w.v_{i}\left(t\right)+c_{1}.rand_{1}.\left(pbest_{i}\left(t\right)-x_{i}\left(t\right)\right)+c_{2}.rand_{2}.\left(gbest_{i}\left(t\right)-x_{i}\left(t\right)\right)$$

(26) $$x_{i}\left(t+1\right)=x_{i}\left(t\right)+v_{i}\left(t+1\right)\;(i=1,\ldots N)$$

where $v_{i}$ and $x_{i}$ resemble the velocity and position of a particle; w denotes the inertia weight which can control the impact of last velocities; and *c*_1_ and *c*_2_ represent the relative impact of the social and cognitive components [91]. A simple flowchart of the PSO approach is illustrated in Figure 4.

#### 2.3.3. Coupled Simulated Annealing

**Figure 5:**
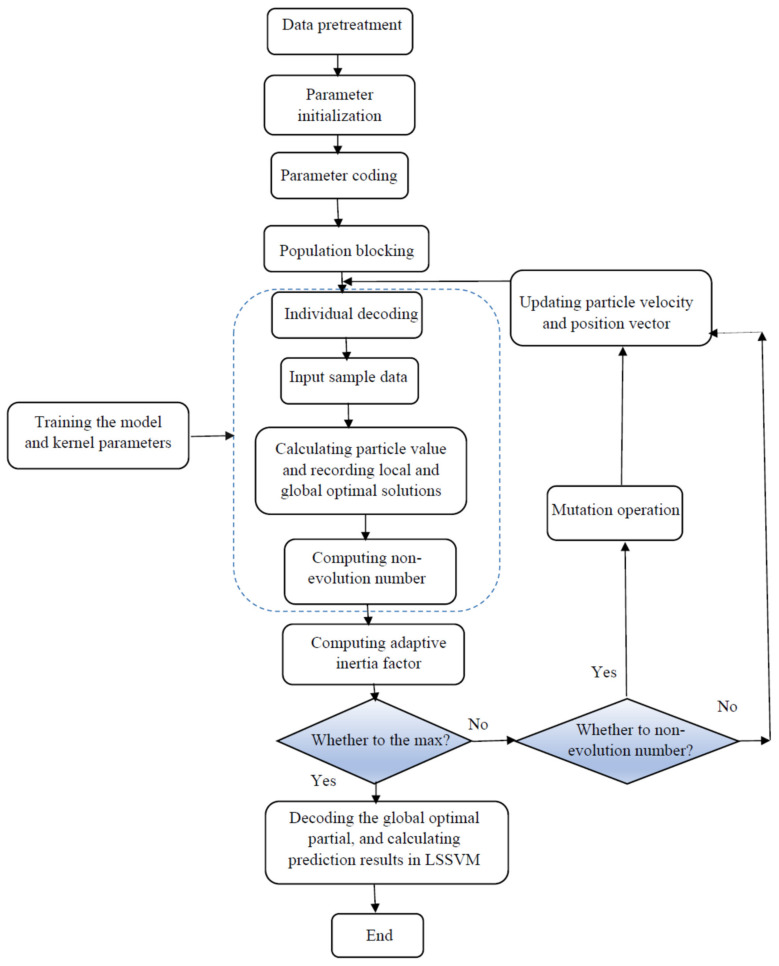
Flowchart of the least square support vector machine—coupled simulated annealing (LSSVM CSA) strategy employed in the current research.

An upgraded form of simulated annealing (SA) is the coupled simulated annealing (CSA), which enhances precision of SA without considerable reduction in the convergence speed. SA has ability to move from the present solution to a worse solution to escape from the local optimum point. During the process, the probability of occurrence of such a movement reduces. The CSA has been suggested in several theoretical and practical optimization cases for easier escape from local optimum so that the precision of the optimization solution improves without unwanted impact on the convergence speed. The major difference between CSA and SA is the probability of acceptance. Suykens summarized the basic principles of CSA to avoid the local optimum for nonconvex problems [92]. More details can be found in the literature [93]. The methodology of the LSSVM-CSA optimization is presented in Figure 5.

#### 2.3.4. Imperialistic Competitive Algorithm

Imperialistic competitive algorithm (ICA) is a new social counterpart approach, which was inspired from the GA algorithm. Atashpaz–Gargari and Lucas introduced this social approach for the first time. ICA shows an excellent ability of detecting the global optimum [94,95]. This algorithm usually uses three popular terminologies; including countries, decade, and cost function. Cost function represents an equation for optimization; decade shows individual iteration; and countries stand for the chromosomes counterparts in the GA algorithm. The countries, which have the least values in cost function, are assigned as the imperialists, and the others are termed as the colonies. The main operators are revolution, competition, and assimilation. The colonies movement toward the imperialist is created by assimilation. The revolution operator changes the location of countries. The mentioned operator controls the process to avoid local minima and improve the ability for finding the best solution. For the assimilation and revolution time, a greater imperialist value than the colony cost function may result in the change of position between the imperialist and colonies. The imperialist competition is defined as taking the colonies’ possession and control of other empires. 

The aforementioned competition can be determined as total cost function (TC) consequence, which is described by the following expression [96,97]:

(27) $$TC_{n}=Cost\left(imprialist_{n}\right)+\xi mean\left\{Cost\left(coloniesofempire_{n}\right)\right\}$$

where ξ stands for the colonies contribution coefficient in TC. The normalization of Equation (27) is expressed as follows:

(28) $$NTC_{n}=TC_{n}-\max\left\{TC_{i}\right\}$$

where NTC refers to the normalized TC. The possession probability for each empire can be determined by the following relationship:

(29) $$PP_{n}=\left|\frac{NTC_{n}}{\sum_{i=1}^{N_{imp}}NTC_{i}}\right|$$

In Equation (29), the imperialists size and possession probability are shown by *N_imp_* and *PP_n_,* respectively. There is similarity between empire selection in ICA and GA. However, the common selection approach such as roulette wheel is applicable in the ICA selection because it does not require the cumulative distribution. The probability vector of *P* is determined as follows:

(30) $$P={\left[PP_{i}\right]}_{l\times N_{imp}}$$

A random number vector and combinatorial vector are obtained as follows:

(31) $$R={\left[r_{i}\right]}_{l\times N_{imp}}$$

(32) $$D=P-R={\left[{\left(pp-r\right)}_{i}\right]}_{l\times N_{imp}}$$

**Figure 6:**
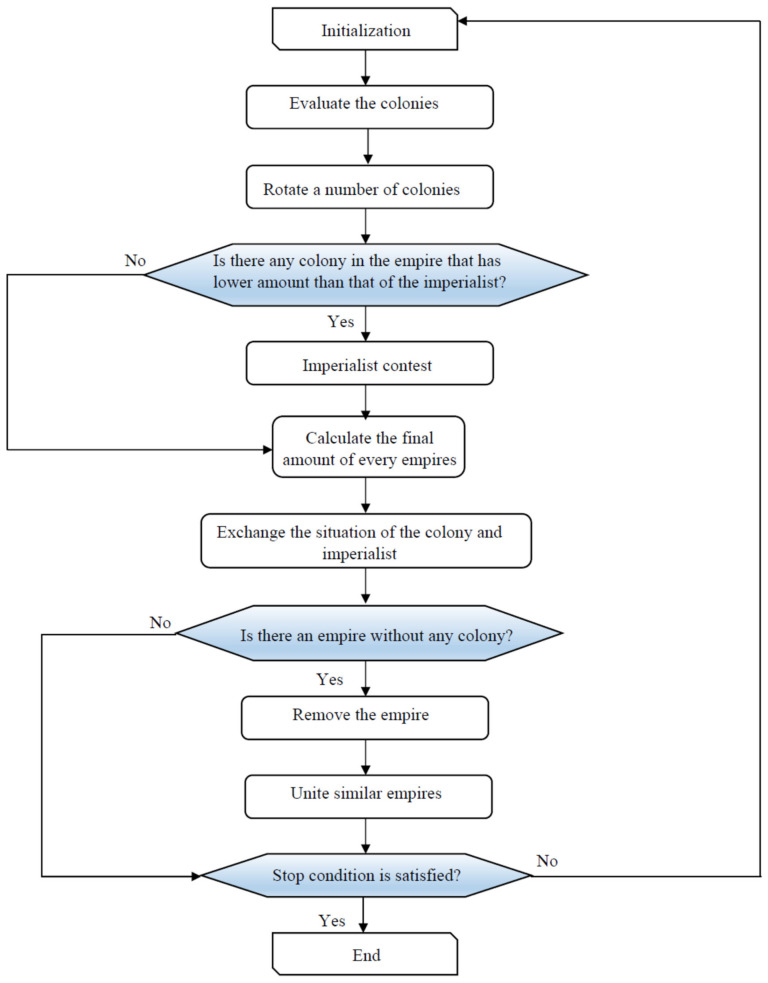
Flowchart to implement imperialist competitive algorithm (ICA).

In this case, the objective is that the pertinent indices of *D* should be maximized. The method of ICA optimization is shown in Figure 6.

#### 2.3.5. Ant Colony Optimization

One of effective population-based algorithms is ant colony optimization (ACO), which was developed based on Dorigo’s work [98]. Searching the least distance between the food and nest is known as the main idea of development of ACO algorithm. The ants’ population uses a chemical component, called pheromone as a footprint, to simulate the best way between the food and nest [99,100]. This algorithm is employed for the discrete path. Hence, the composite probabilistic modeling from Gaussian distribution should be implemented as probable solutions. In this case, the pheromone approach is applicable to modeling continuous paths. The probabilistic strategy obtains the best solution based on comparison of results with previous step. In order to find the solution vector of *x*, it is necessary to minimize the objective function (OF). The steps below express the computations in the ACO algorithm [101,102,103]:

1. For *N* number of selected random solutions, the OF should be determined.
2. The best and worst initial solutions are denoted by *x*_1_ and *x_N_*, respectively, which are necessary to organize the solution.
3. The following expression is used to assign a weight for each individual solution:

(33) $$u_{i}\propto \frac{1}{\sqrt{2\pi }\alpha N}\exp\left(-\frac{1}{2}{\left(\frac{i-1}{\alpha N}\right)}^{2}\right)$$

For all weights, the following relationship should hold:

(34) $$\overset{N}{\underset{i=1}{\sum }}u_{i}=1$$

4. Then, the Gaussian composite probabilistic modeling is constructed based on the following expression:

(35) $$G^{j}\left(x\left[j\right]\right)=\overset{N}{\underset{i=1}{\sum }}u_{i}N\left(x\left[j\right];\mu_{i}\left[j\right],\sigma_{i}\left[j\right]\right)$$

(36) $$N\left(x;\mu ,\sigma \right)=\frac{1}{\sqrt{2\pi }\sigma }\exp\left(-\frac{1}{2}{\left(\frac{x-\mu }{\sigma }\right)}^{2}\right)$$

where *x[j]* and $x\left[j\right]$ are the *j^th^* component of the *x* as a solution and a decision parameter, respectively. The following equations represent the average parameter and standard deviation:

(37) $$\mu_{i}\left[j\right]=x_{i}\left[j\right]$$

(38) $$\sigma_{i}\left[j\right]=\frac{\xi }{N-1}\overset{N}{\underset{i^{\prime }=1}{\sum }}\left|x_{i}\left[j\right]-x_{i^{\prime }}\left[j\right]\right|$$

where ξ is a real positive value, which indicates the exploration–exploitation balance.

5. The *M* samples as the solution offspring are created by the multidimensional model, as given below:

g = (G^1^,G^2^,…,G^ns^) (39)

6. The *M* offspring and *n* best solution are chosen.
7. The termination criterion is checked.

**Figure 7:**
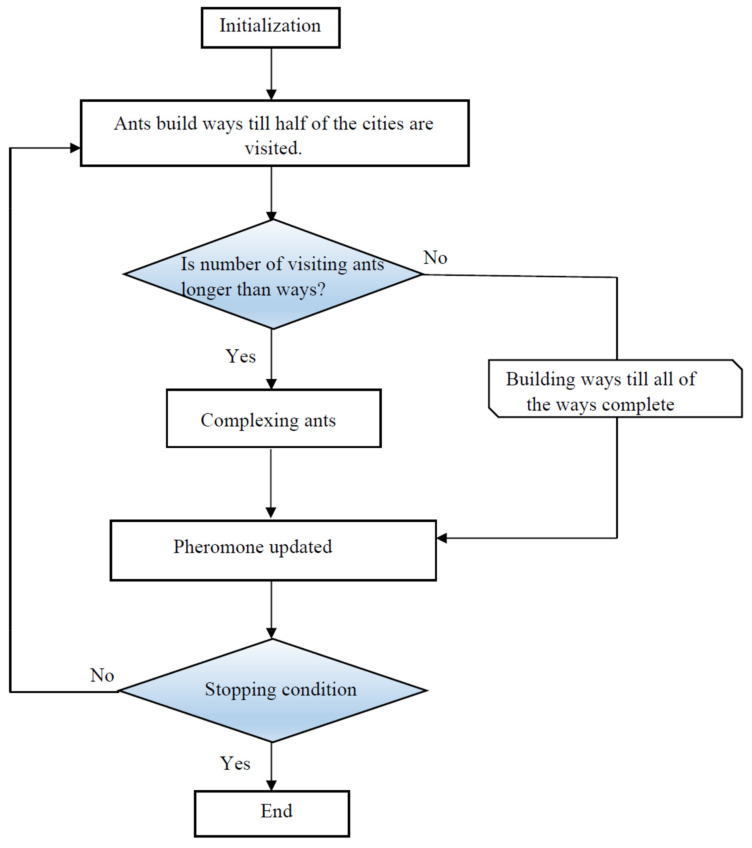
Flowchart of the ant colony optimization (ACO) algorithm.

A schematic of the ACO approach is described in Figure 7. 

## 3. Results and Discussion

In a computational and modeling work, the modeling outputs are compared to experimental data in order to assess the model performance. The statistical indexes, including root mean square error (RMSE), R-squared (R^2^), standard deviation (SD), average percent relative error (APRE, %), and average absolute percent relative error (AAPRE, %), defined below, are employed in this work for model evaluation:

(40) $$\mathrm{SD}=\sqrt{\frac{1}{N-1}\sum_{i=1}^{N}{\left(\frac{X_{i}^{actual}-X_{i}^{predicted}}{X_{i}^{actual}}\right)}^{2}}$$

(41) $$R-\mathrm{squared}\;(R^{2})=1-\frac{\sum_{i=1}^{N}{\left(X_{i}^{\mathrm{actual}}-X_{i}^{\mathrm{predicted}}\right)}^{2}}{\sum_{i=1}^{N}{\left(X_{i}^{\mathrm{actual}}-\bar{X^{\mathrm{actual}}}\right)}^{2}}$$

(42) $$\mathrm{APRE}=\frac{100}{N}\sum_{i=1}^{N}(\frac{X_{i}^{actual}-X_{i}^{predicted}}{X_{i}^{actual}})$$

(43) $$\mathrm{AAPRE}=\frac{100}{N}\sum_{i=1}^{N}|\frac{X_{i}^{actual}-X_{i}^{predicted}}{X_{i}^{actual}}|$$

(44) $$\mathrm{RMSE}=\sqrt{\frac{1}{N}\sum_{i=1}^{N}({\left(X_{i}^{exp.}-X_{i}^{predicted}\right)}^{2})}$$

In the current study, 252 data points from 3 nanocomposites are gathered to properly simulate asphaltene adsorption onto nanocomposites with various characteristics under different operating conditions. Ten models are developed, in which MLP is optimized using BR, LM, SCG, and CGF; RBF is optimized thorough ACO, ICA, GA, and PSO; LSSVM is optimized with CSA; and the last one is GMDH. The computational time of each model employed in this study is presented in Table 4. In prediction of asphaltene adsorption, the low values of SD, RMSE, AAPRE, and APRE and high values of R^2^ for the training and testing phases imply the accuracy and general applicability of the proposed models. Overall, on the basis of the results shown in Table 4, the accuracy of the suggested models can be ranked as follows:

RBF-ACO > LSSVM-CSA > MLP-BR > MLP-LM > RBF-ICA > MLP-SCG > MLP-CGF > RBF-GA > RBF-PSO > GMDH

**Figure 8:**
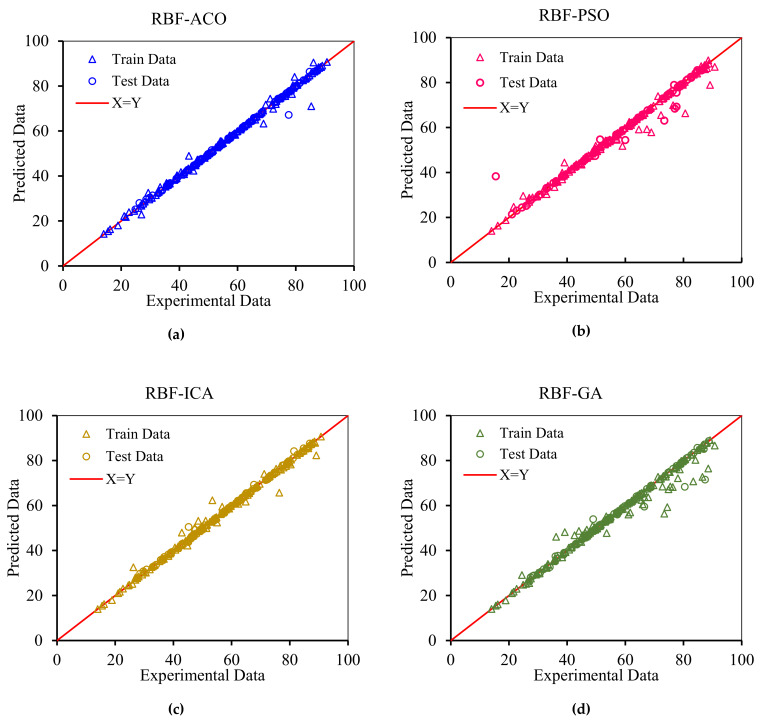

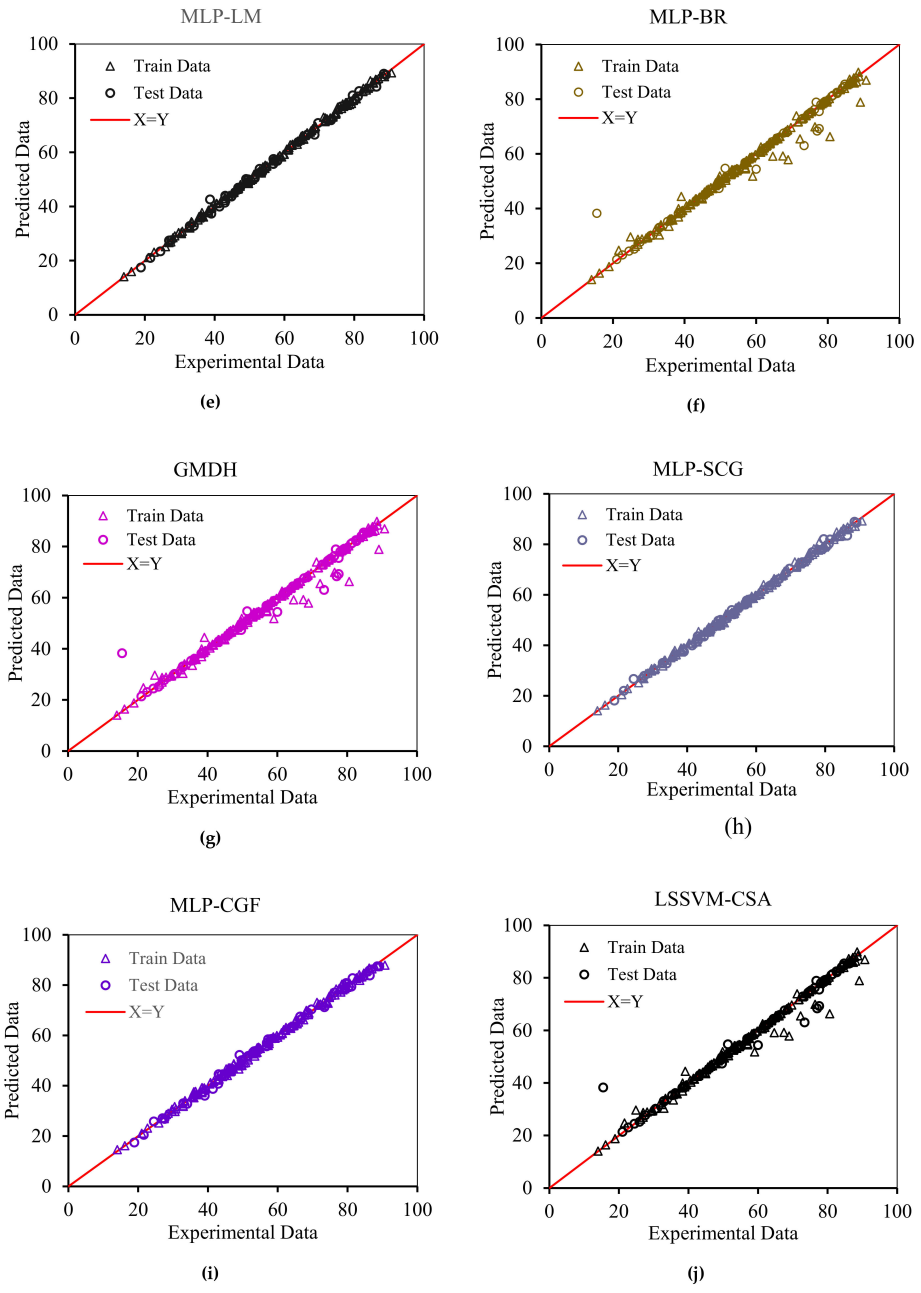
Cross plots for the asphaltenes adsorption models presented in this study for both training and testing subsets: (**a**) RBF-ACO, (**b**) RBF-PSO, (**c**) RBF-ICA, (**d**) RBF-GA, (**e**) MLP-LM, (**f**) MLP-BR, (**g**) GMDH, (**h**) MLP-SCG, (**i**) MLP-CGF, and (**j**) LSSVM-CSA.

**Figure 9:**
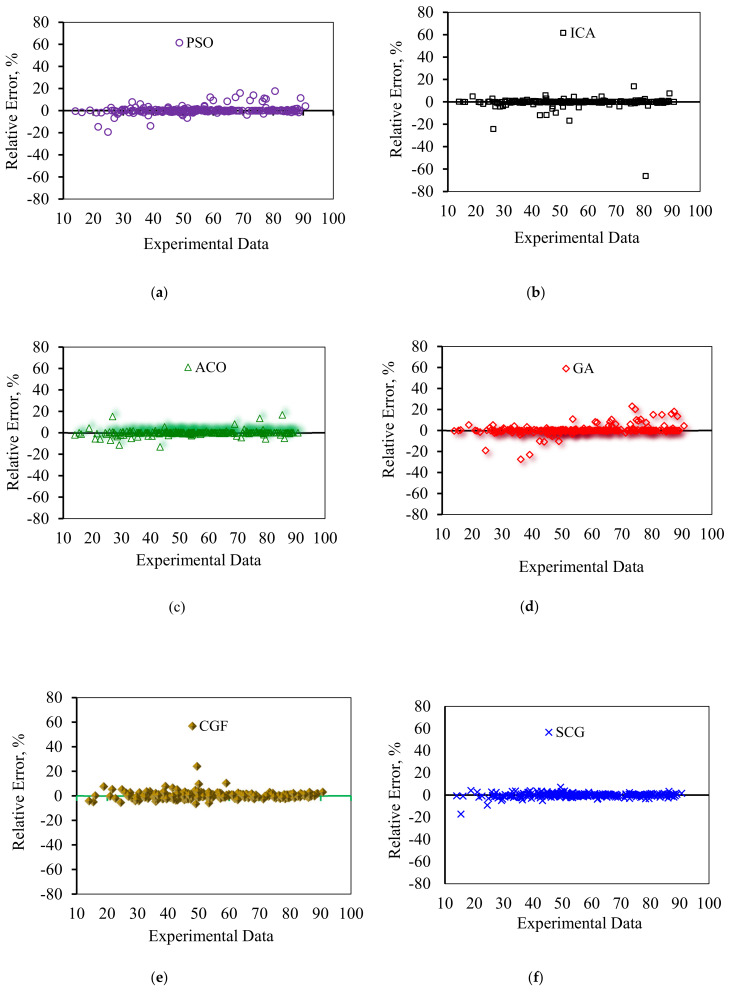

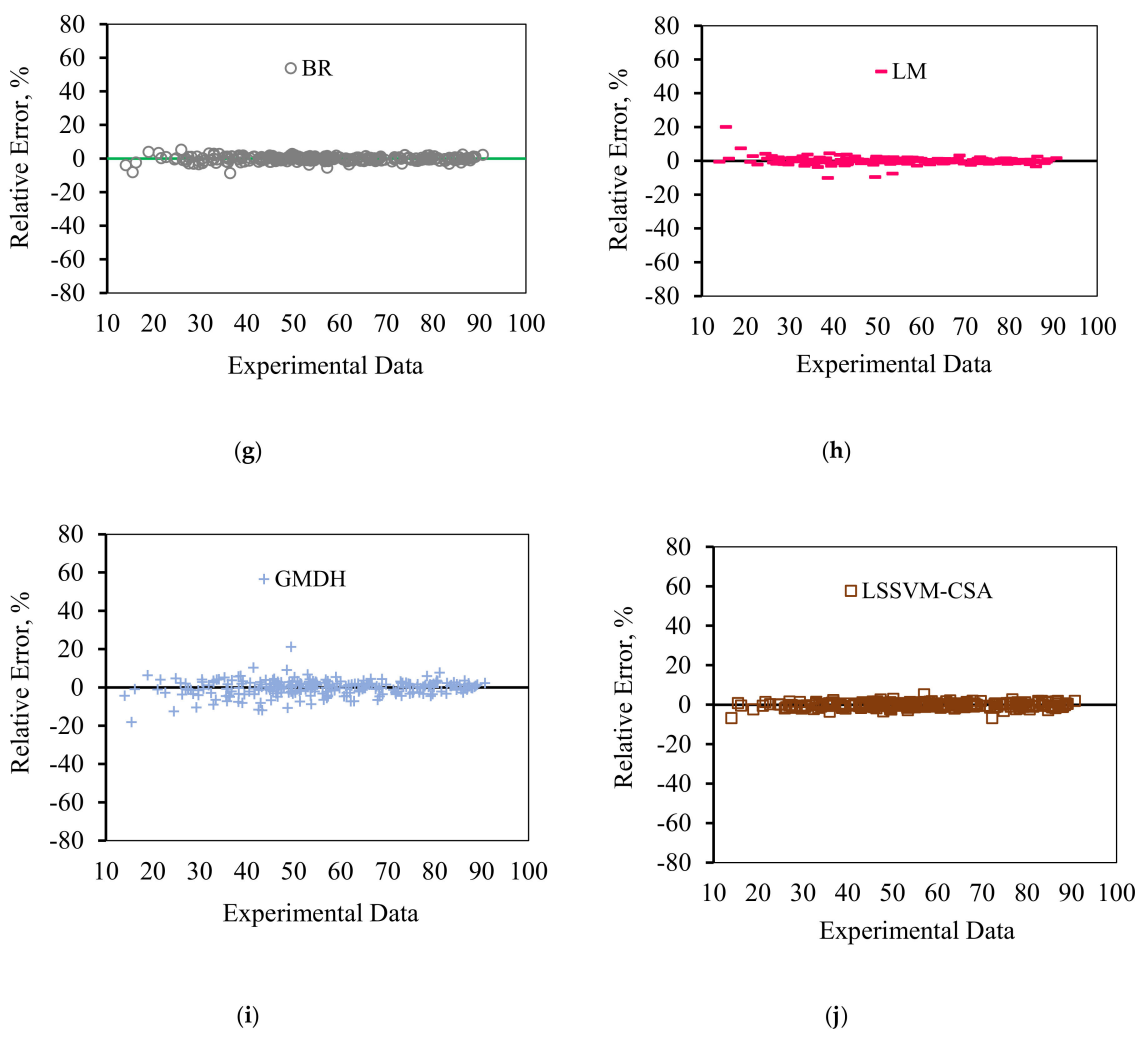
Error distribution for the proposed asphaltenes adsorption models versus experimental data: (**a**) PSO, (**b**) ICA, (**c**) ACO, (**d**) GA, (**e**) CGF, (**f**) SCG, (**g**) BR, (**h**) LM, (**i**) GMDH, and (**j**) LSSVM-CSA.

In order to graphically confirm the accuracy of the models, the cross plots of anticipated data versus experimental data as well as error distribution for the testing and training data are plotted in Figure 8 and Figure 9, respectively. Figure 8 shows an excellent match between the model predications and the experimental asphaltene adsorption onto the surface of nanocomposites. Experimental asphaltene adsorption data are mostly located very close to *y = x* line. Figure 9 demonstrates that a majority of data points of all models are placed at (or close to) zero error line which, in turn, verifies the consistency between the predicated and real data points. For most of the models, the maximum relative error between the predicated and experimental data is around 30%. However, in ICA the maximum error is around 70%, showing higher deviations in prediction.

**Figure 10:**
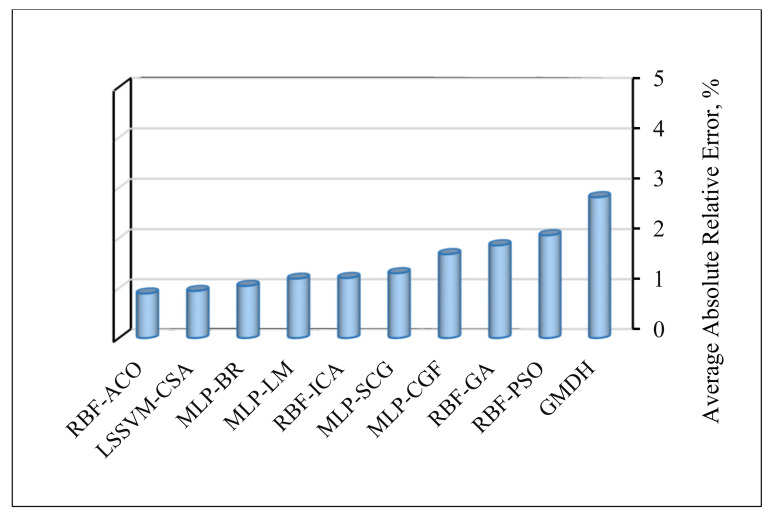
Average absolute percent relative error (AAPRE) for the asphaltenes adsorption models proposed in this research.

Although all of the proposed models exhibit a very good match with the experimental data, it is constructive to identify the best algorithm in this research in terms of precision and reliability. The magnitudes of average absolute relative error (AARE), which is the most vital criterion for the assessment of model performance, are presented in Figure 10. Among the ten proposed models, RBF-ACO and LSSVM-CSA with AAPRE% values under 1% appear the most accurate, whereas the GMDH has the least accuracy. However, one major asset of GMDH is that it creates a visual relationship between the inputs and output; it can be also easily applied.

**Figure 11:**
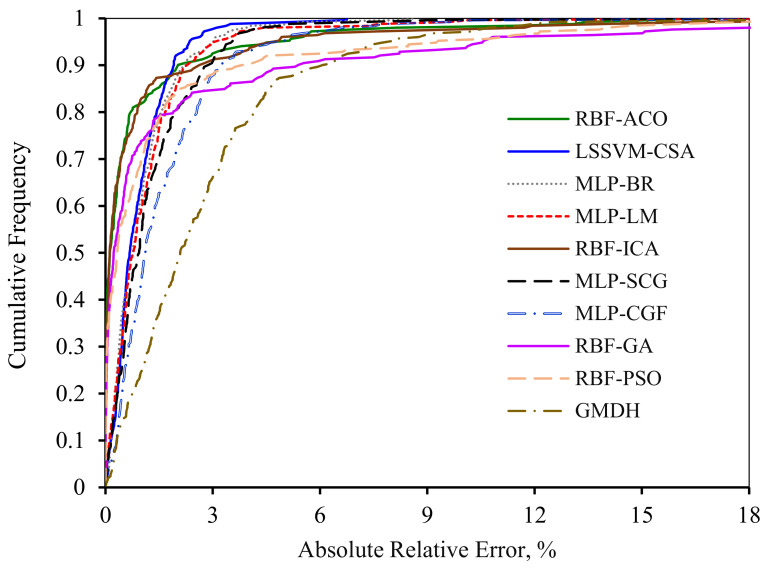
Cumulative frequency curve of introduced models of asphaltenes adsorption as a function of absolute relative error.

In order to make a graphical comparison among all of the models, the variations of absolute relative error in terms of data points cumulative frequency for the entire models are illustrated in Figure 11. It should be mentioned that the precision and robustness of the models increase as the graphs become closer to the y axis. It is obvious from Figure 11 that both RBF-ICA and RBF-ACO have a great accuracy as these models can predict 85% of the data with an absolute relative error less than 1%. Other deterministic tools estimate 70% of the data points with an absolute relative error around 1%. However, both MLP-CGF and GMDH techniqes have the highest deviation such that they can predict 60% and 30% of the data points with an absolute relative error less than 1%, respectively.

**Figure 12:**
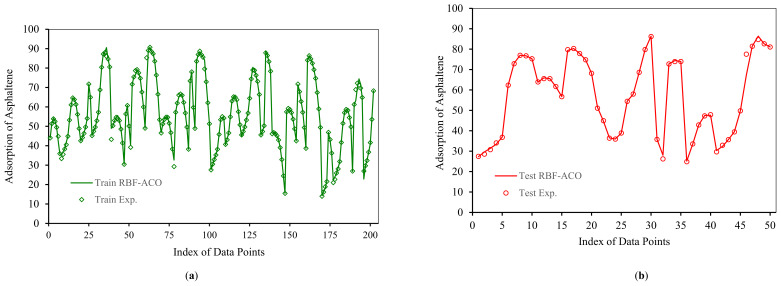
Comparison between predicated and experimental asphaltenes adsorption by RBF-ACO for (**a**) Train; (**b**) Test.

According to Figure 10 and Figure 11, RBF-ACO has the best performance among all algorithms. The error indexes for RBF-ACO are APRE% = −0.08, AAPRE% = 0.89, RMSE = 1.42, and *R^2^* = 0.9937. One practical tool for evaluation of a model performance is plotting actual versus predicted data, as depicted in Figure 12 for RBF-ACO. As it is evident from Figure 12, great agreement between RBF-ACO model’s predictions and the experimental asphaltene adsorption data is noticed for the training and testing phases. It again indicates a high degree of accuracy attained from this model.

**Figure 13:**
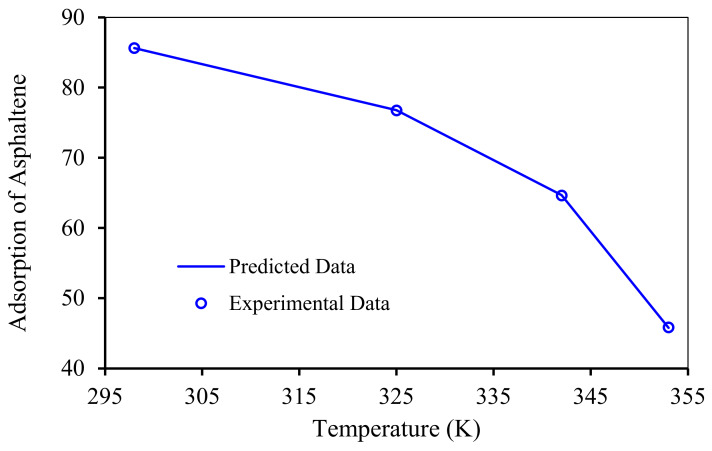
Experimental and predicted data by the RBF-ACO model for adsorption of asphaltenes by NiO/ZSM-5 at different temperatures and a pH of 4.8.

The accuracy of RBF-ACO includes all range of experimental conditions. For example, Figure 13 shows an excellent match between the RBF-ACO model fit and the experimental asphaltene adsorption within the temperature range of 295 to 355 K. Higher temperatures negatively affect the asphaltene uptake by NPs and nanocomposites, as shown in Figure 13, which is in agreement with published experimental investigations [58,104]. In fact, higher temperatures impact NPs and asphaltene aggregation state as well as crude oil properties. It is worth mentioning that thermodynamic studies should be conducted in order to better scrutinize effects of temperature.

**Figure 14:**
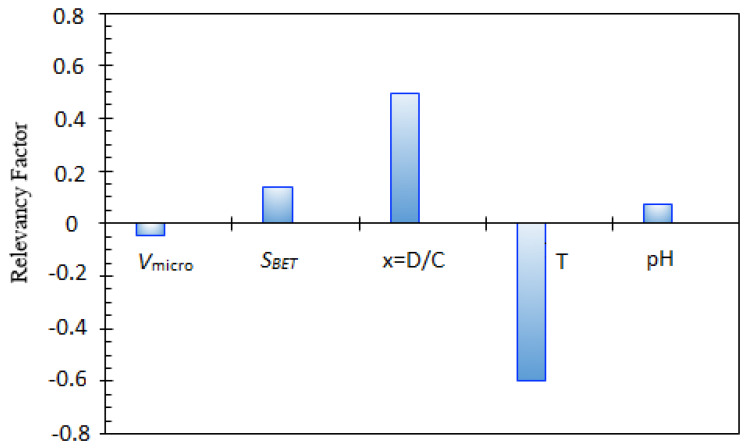
Relative contribution of various input variables in asphaltenes adsorption.

A key statistical analysis implemented in the current work is sensitivity analysis. This method is used to quantify the impacts of the type of nanocomposite, pH, D/C_0_, and temperature on asphaltenes adsorption. The relevancy factor, which is the main parameter in this method, is expressed by the following relationship [68,88]:

(45) $$r=\frac{\sum_{i=1}^{n}(X_{k,i}-\bar{X_{k}})\left(Z_{i}-\bar{Z}\right)}{\sqrt{\sum_{i=1}^{n}{\left(X_{k,i}-\bar{X_{k}}\right)}^{2}\sum_{i=1}^{n}(Z_{i}-{\bar{Z)}}^{2}}}$$

where $\;X_{k,i}$, $\bar{X_{k}}$, $Z_{i},\;$and $\bar{Z}$ introduce the *k^th^* input, input averages, target parameter(s), and its average, respectively. Figure 14 shows this parameter for each input variable. It follows that volume of the micropore, S_BET_, pH, and D/C_0_ display a straight-line relationship with asphaltene adsorption from the model oil by nanocomposites. Moreover, increasing temperature, which is the most effective parameter, decreases asphaltenes adsorption from the model solutions as proved by experimental studies. It can be concluded that the two most influential parameters are the temperature and D/C_0_. It was found that an increase in the efficiency of asphaltene adsorption is experienced as D/C_0_ increases. Furthermore, at low pH, electrostatic forces cause attraction between adsorbent with positive surface charge and asphaltene with negative surface charge. An increase in pH causes repulsion forces between asphaltenes with negative surface charge and OH− ions which, in turn, leads to a decline in the asphaltene adsorption process [62].

Various algorithms and procedures have been proposed for exclusion and determination of outliers. In this study, the Leverage strategy is applied to scrutinize the experimental data. This approach determines the model deviations from experimental data points [105,106]. In this method, Hat matrix is computed as follows [106,107,108,109]:

(46) $$H=A{\left(A^{T}A\right)}^{-1}A^{T}$$

where *A* denotes an *a × b* matrix in which *b* and *a* are the number of the model’s parameters and samples, respectively. Another important parameter in this method is the leverage limit, as defined below:

(47) $$H^{*}=3\left(b+1\right)/a$$

**Figure 15:**
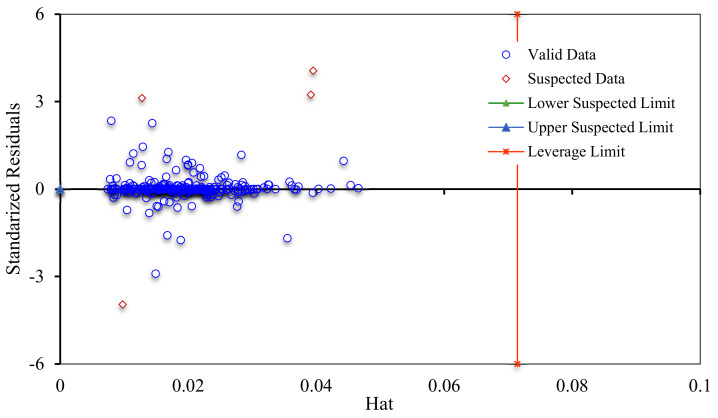
William’s plot for determining the applicability domain and suspected data points of asphaltenes adsorption onto NPs.

Hat values are plotted against standardized residuals (SR: deviations between experimental data and modeling results); the resultant figure is named William’s plot. In this strategy, the experimental data have a good quality and the model is statistically valid, if a majority of the data points are located in the feasibility domain of the RBF-ACO model (0 ≤ hat ≤ 0.071 and −3 ≤ SR ≤ 3). Figure 15 demonstrates that only four experimental asphaltene adsorption data points fall in the suspected area; thus, the collected experimental dataset is sufficiently reliable to train and test the models; the RBF-ACO model is also statistically acceptable.

## 4. Conclusions

Asphaltene precipitation is one of the most problematic issues in many oil reservoirs worldwide. A novel method for the treatment of asphaltenes, i.e., NPs-based treatments, has shown an excellent ability in tackling asphaltene-related problems since NPs and nanocomposites have great affinity toward adsorption and removing asphaltenes from crude oils. In this work, artificial intelligence models are developed to forecast adsorption of asphaltenes onto nanocomposites as a function of type of nanocomposites, pH, D/C_0_, and temperature. The presented algorithm outputs are graphically and statistically compared with actual asphaltenes adsorption data. Deviation between the model fit and experiments is described using different statistical indexes. It was found that LSSVM, ANN, and GMDH optimized by LM, BR, CGF, SCG, GA, PSO, CSA, ICA, and ACO sufficiently simulate asphaltenes adsorption data onto nanocomposites. The radial basis function neural network and the least-squares support vector machine, which are optimized by ant colony optimization and coupled simulated annealing, respectively, exhibit the best performance. RBF-ACO and LSSVM-CSA have the most accuracy with AARE% values of 0.89% and 0.94%, respectively. In addition, the impact of the different input variables on asphaltenes adsorption is analyzed. The temperature and the pore volume of the nanocomposites have the most and the least influence on asphaltenes adsorption, respectively. Lastly, the leverage strategy is employed to assess the quality of collected data, confirming the reliability of the data and the proposed RBF-ACO model. 

## Figures and Tables

**Table 1:** Summary of nanocomposites properties used in this study.

References	Nanoparticles	BET Surface Area (m^2^/g)	Pore Volume (cm^3^/g)	Volume of Micropore (cm^3^/g)	Method of Synthesis	Other Properties
[61]	NiO/SAPO-5 composite	304	0.252	0.122	SAPO-5 was synthesized by means of hydrothermal method. NiO/SAPO-5 composite was synthesized via an eco-friendly template (tetramethylguanidine, TMG).	Size of NiO was 20-35 in nm. NiO/SAPO-5 had a mean particle size of NiO 27.5 ± 7.5 nm.
[62]	NiO/ZSM-5 nanocomposite	348	0.126	0.103	A green bio-based cadaverine template created from decarboxylation of amino acids was employed to synthesize NiO/ZSM-5.	Percentage of crystallinity was 89%. Size of NiO was 20-35 nm.
[64]	NiO/AlPO-5 nanocomposite	298	0.252	0.116	AlPO-5 powder was synthesized through the hydrothermal procedure. NiO/AlPO-5 nanocomposite was synthesized by using green TMG.	Size of NiO was 20-35 nm.

**Table 2:** Summary of experiments conditions.

References	Nanoparticles	Oil Properties	Adsorbent–Oil Ratio	Model Solutions or Crude Oil	Asphaltenes Extraction Method
[61]	NiO/SAPO-5 composite	Asphaltene content was 11.5 wt%; API gravity was 26.8; total acid number of oil was 0.13 mg KOH/g	Experiments were conducted with a ratio of 10:1 g/(mg/L).	Model oil solution	IP-143
[62]	NiO/ZSM-5 nanocomposite	Asphaltene value content was 11.4 wt %; API gravity was 26.7	Experiments were carried out with a ratio of 10:1 g/(mg/L).	Model oil solution	ASTM D2007-80
[64]	NiO/AlPO-5 nanocomposite	Asphaltene content was 11.5 wt%	Experiments were performed with a ratio of 10:1 g/(mg/L).	Model oil solution	IP-143

**Table 3:** Correlations developed by group method of data handling (GMDH) for predicting the amount of asphaltenes adsorption onto nanocomposites.

$N_{7}=$	$-65.9365+pH\times 25.1354-pH\times X\times 173.589-pH^{2}\times 1.22985+X\times 2254.08-X^{2}\times 11287.3$
$N_{6}$=	$-851.238+T\times 5.66144-T\times X\times 4.89965-T^{2}\times 0.00909235+X\times 2855.01-X^{2}\times 9304.22$
$N_{5}$=	$-67.7102+pH\times 21.3243-pH\times N_{6}\times 0.0777542-pH^{2}\times 1.51952+N_{6}\times 1.46848$
$N_{4}=$	$-939.17+T\times 5.87837-T\times N_{7}\times 0.00862458-T^{2}\times 0.00911196+N_{7}\times 3.64439+N_{7}{}^{2}\times 0.00195994$
$N_{3}=$	$0.695476+N_{4}\times 1.37283+N_{4}\times N_{5}\times 0.0119274-N_{4}{}^{2}\times 0.0117821-N_{5}\times 0.391564$
$N_{2}$=	$356.011-V\times 6419.24-V\times N_{3}\times 1.39521+V^{2}\times 28923.4+N_{3}\times 1.09722+N_{3}{}^{2}\times 0.000452982$
$N_{1}$=	$0.801253+X\times N_{2}\times 2.56285-X^{2}\times 1152.67+N_{2}\times 1.00765-N_{2}{}^{2}\times 0.00156164$
Y=	$4.1312-pH\times 1.9062+pH^{2}\times 0.142768+N_{1}\times 1.04536-N_{1}{}^{2}\times 0.000326059$

**Table 4:** Magnitudes of root mean square error (RMSE), standard deviation (SD), average percent relative error (APRE), average absolute percent relative error (AAPRE), computational time, and coefficient of determination (R^2^) for all the proposed models for prediction of asphaltenes adsorption.

Model		APRE, %	AAPRE, %	RMSE	R^2^	SD	Computation Time (min)
RBF-ACO	Train	−0.09	0.90	1.39	0.9937	0.00061	120
	Test	−0.03	0.84	1.53	0.9939	0.00053	
	Total	−0.08	0.89	1.42	0.9937	0.00059	
LSSVM-CSA	Train	−0.07	0.95	0.67	0.9986	0.00018	20
	Test	−0.25	0.91	0.64	0.9988	0.00013	
	Total	−0.11	0.94	0.66	0.9986	0.00017	
MLP-BR	Train	−0.01	0.95	0.62	0.9989	0.00013	15
	Test	−0.21	1.34	1.00	0.9963	0.00039	
	Total	−0.10	1.04	0.72	0.9984	0.00023	
MLP-LM	Train	−0.07	0.83	0.56	0.999	0.00010	10
	Test	0.65	2.08	1.22	0.9963	0.00064	
	Total	0.017	1.19	0.85	0.9978	0.00045	
RBF-ICA	Train	−0.49	1.27	4.01	0.9536	0.00300	165
	Test	−0.63	0.92	0.99	0.9972	8.96E-05	
	Total	−0.52	1.20	3.61	0.9618	0.00249	
MLP-SCG	Train	−0.02	1.07	0.73	0.9983	0.00017	10
	Test	0.34	1.73	1.04	0.9972	0.00045	
	Total	−0.07	1.30	0.87	0.9977	0.00041	
MLP-CGF	Train	−0.07	1.31	0.83	0.9977	0.00023	15
	Test	0.69	2.36	1.37	0.9953	0.00077	
	Total	0.15	1.68	1.30	0.9948	0.00072	
RBF-GA	Train	0.50	1.90	2.9	0.9753	0.00225	180
	Test	0.57	1.66	3.10	0.9745	0.00170	
	Total	0.51	1.85	2.94	0.9752	0.00214	
RBF-PSO	Train	0.28	1.42	2.01	0.9861	0.00119	220
	Test	−2.29	4.95	4.29	0.9628	0.04571	
	Total	−0.18	2.05	2.55	0.9805	0.00988	
GMDH	Train	−0.14	2.84	1.92	0.9863	0.00167	20
	Test	−0.26	2.66	1.95	0.9819	0.00120	
	Total	−0.17	2.81	1.92	0.9853	0.00157	

## Nomenclatures

*A a × b*	matrix in Hat matrix
*a*	Number of algorithm samples
AAPRE	Average absolute percent relative error
ACO	Ant colony optimization
ANN	Artificial neural network
ANFIS	Adaptive neuro-fuzzy inference system
APRE	Average percent relative error
*b*	Number of algorithm parameters
BR	Bayesian regularization
c_1_	Relative impact of the social components
c_2_	Relative impact of the cognitive components
CGF	Conjugate gradient with Fletcher- Reeves updates
CSA	Coupled simulated annealing
D	Combinatorial vector
g_best_	Best global position
GA	Genetic algorithm
GMDH	Group method of data handling
H	Hat matrix
$H^{*}$	Leverage limit
ICA	Imperialistic competitive algorithm
ISC	In situ combustion
LIBS	Laser-Induced Breakdown Spectroscopy
LM	Levenberg-Marquardt
LSSVM	Least-squares support vector machine
MLP	Multilayer perceptron
MW	Molecular weight
N_imp_	Imperialists size
NPs	Nanoparticles
NTC	Normalized TC
OF	Objective function
p_best_	Best visited position
PNN	Polynomial neural network
PP_n_	Imperialists possession probability
PSO	Particle swarm optimization
R	A random number vector
r	Relevancy factor
R^2^	Coefficient of determination
RBF	Radial basis function
RMSE	Root-mean-square error
S_BET_	BET surface area
SA	Simulated annealing
SCG	Scaled conjugate gradient
SD	Standard deviation
TC	Total cost value
$v_{i}$	Velocity of a particle
$X_{k,i}$	i-th input value of the k-th input parameter
$\bar{X_{k}}$	Average value for the k-th input
$Z_{i}$	i-th output value
$\bar{Z}$	Average value for output parameter

## Greek Letters

γ	Regularization parameter
ω	Weight matrix
$\varepsilon_{i}$	Slack variable
$\varphi \left(X_{i}\right)$	Kernel function
$\emptyset_{i}$	Radial basis function
α_K_	Lagrangian multipliers
*σ*	Gaussian spread
